# Agency as a mediator in the pathway from transactional sex to HIV among pregnant women in Swaziland: a multigroup path analysis

**DOI:** 10.7448/IAS.20.1.21554

**Published:** 2017-07-18

**Authors:** Rebecca Fielding-Miller, Kristin L Dunkle, Craig Hadley, Hannah LF Cooper, Michael Windle

**Affiliations:** ^a^ Division of Global Public Health, Center on Gender Equity and Health, University of California, San Diego, CA, USA; ^b^ Gender and Health Division, South African Medical Research Council, Pretoria, South Africa; ^c^ Department of Anthropology, Emory University, Atlanta, GA, USA; ^d^ Department of Behavioral Sciences and Health Education, Rollins School of Public Health, Emory University, Atlanta, GA, USA

**Keywords:** transactional sex, Swaziland, structural equation modeling, social status, HIV/AIDS, agency, cultural consensus modeling

## Abstract

**Introduction**: Transactional sex is a structural driver of HIV for women and girls in sub-Saharan Africa. In transactional relationships, sexual and economic obligations intertwine and may have positive and negative effects on women’s financial standing and social status. We conducted a clinic-based survey with pregnant women in Swaziland using a locally validated transactional sex scale to measure the association between subjective social status, transactional sex, and HIV status, and to assess whether this association differed according to a woman’s agency within her relationship.

**Methods**: We recruited a convenience sample of 406 pregnant women at one rural and one urban public antenatal clinic in Swaziland and administered a behavioural survey that was linked to participant HIV status using clinic records. We then conducted a multigroup path analysis to test three hypotheses: (1) that more engagement in transactional sex is associated with decreased condom use and increased subjective social status; (2) that subjective social status mediates the relationship between transactional sex and HIV status; and (3) that these relationships are different across groups according to whether or not a woman reported any indicator of constrained agency within her relationship.

**Results**: The amount and value of material goods received from a sexual partner was significantly and positively associated with higher subjective social status among all participants. As the amount of material goods received from a partner increased, women who reported no indicators of constrained agency were less likely to use condoms. Conversely, there was no relationship between transactional sex and condom use among women who reported any indicator of constrained relationship agency. Among women who reported any indicator of constrained agency, HIV was significantly associated with lower subjective social status.

**Conclusions**: Relationship agency likely plays a key role in determining which mechanisms create HIV risk for women in transactional relationships. Interventions to mitigate these risks must address social forces that penalize women who engage in sexual relationships as well as structural drivers of gendered economic disparity that reduce women’s agency within their sexual and romantic relationships.

## Introduction

While the risks and motives may overlap, transactional sex - frequently defined as “the exchange of money or gifts for sex” [1] - is distinct from sex work. Sex workers and their clients typically perceive their encounter as commercial, and female sex workers face a distinct set of risks as a direct result of the stigma associated with sex work [2,3]. The type of relationships described by the term “transactional sex” is more nebulous. Women may engage in a transactional relationship for basic survival needs or fashionable consumer goods, to support their families or funnel money to a poorer boyfriend, or because they enjoy having a high-status partner [4–10].

Women who engage in transactional sex are 50% more likely to be living with HIV than women who do not identify their relationship as transactional [11,12]. While transactional sex is typically considered at the individual level, individual women’s risks are influenced by forces at every level of the social ecology, including personal preferences, partner characteristics, violence within the relationship, social pressure from family and friends, local and international laws, and societal gender norms [10,12–15].

Understanding the pathways that drive HIV risk in transactional relationships requires considering how women’s motives influence and are influenced by their romantic and sexual relationship as well as broader social forces. Women’s agency is perhaps best understood as their ability to evaluate and navigate the realities of both their relationships and the broader social landscape in a way that allows them to choose and achieve their own preferred outcome [16,17]. One study from South Africa found that while agency is likely an important mediator of risk for young women who engage in transactional relationships, complex pathways necessitate studies which carefully consider the nuances of how we conceptualize and measure transactional sex, as well as the social landscape within which these relationships exist [18]. The research presented here is part of a larger project that was designed to understand what women hope to gain materially and socially from transactional sex, how the social and material consequences of transactional sex may mediate the association between transactional sex and HIV risk, and whether or not a woman’s agency - in addition to simply the amount of material support she receives from her partner - is a key risk factor within sexual-economic relationships [19,20].

### Conceptual framework

**Figure 1.**
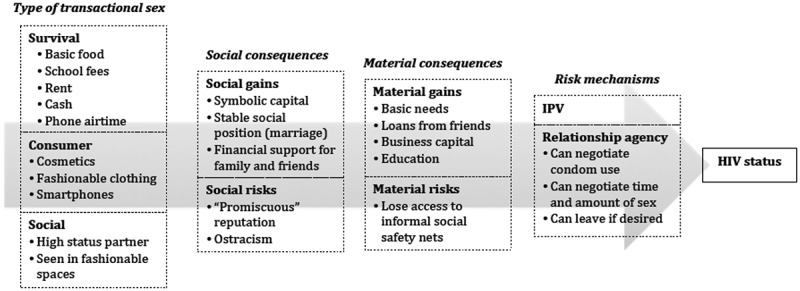
Conceptual framework of transactional sex risk.

Transactional relationships exist within a social landscape. Women’s sexual relationships affect their social status within a community and can generate material gains and losses beyond what they receive from their partner (Figure 1) [5,21,22]. A high-status partner or access to consumer goods may increase a woman’s social capital within her community, particularly if the relationship is long term or they are married [6,7,20,23]. It can also strengthen her social network if she is able to provide for her family and friends through his financial support [4,5,7,10,20]. Friends may be more willing to lend women money in times of crisis if they know she is likely to be able to pay them back [20,24].

Despite the potential gains of a high-status partner, being perceived as promiscuous or materialistic increases the risk that a woman may be cut off from support in times of need [5,6,25]. These motives may be especially stigmatized because they are considered incompatible with normative notions of African femininity [26]. If women who are perceived this way are also living with HIV, they may be accused of bringing misfortune on themselves through deviant behaviour and face the loss of both social and material resources [21,27].

A woman’s degree of agency within her relationship may be just as important to her risk as whether or not her partner provides her with financial support [20]. Despite its significant association with HIV, the act of exchanging material goods for sex or receiving financial support from a male partner alone is not risky. For example, women who acknowledge the utilitarian nature of their relationships may feel that they are at higher risk of HIV and other STIs and be more likely to negotiate condom use [28], although women who lack agency because they are financially dependent may be less able to do so, or to exit a relationship in which they no longer feel safe [29,30].

### Current study

Qualitative studies have explored the array of motives and the social landscape in which transactional relationships take place [4–6,10,21,31], suggesting that transactional sex is better understood as a series of continua with “fuzzy borders” rather than a discrete binary [32]. However, we could identify only one quantitative study that attempted to measure the mediators driving the link between HIV and transactional sex [18], and none that measured transactional sex as a spectrum rather than a binary. Nor could we identify any quantitative studies that tested how social status influences the link from transactional sex to a woman’s HIV status, or how these relationships may change according to a woman’s level of agency within her relationship.

We conducted a quantitative study in Swaziland using a culturally validated scale of transactional sex to test how a woman’s social status mediates the risk pathway between transactional sex and HIV status, whether the cumulative social value of items received from a partner influences condom use in the context of HIV risk, and whether or not these relationships vary depending on a woman’s degree of agency within her relationship.

## Methods

### Setting and study context

Swaziland is a small nation in southern Africa with the world’s highest HIV prevalence: a 2010 population level study found that 31% of adults aged 18–49 are currently living with the virus and antenatal surveillance data from the same year suggests that prevalence peaks at 54% for pregnant women aged 30–34 [33,34]. Although technically considered a middle-income country, Swaziland has high income inequality - the majority of Swazis live on less than $1.25 a day while the king enjoys an annual household budget of US $61 million [35–37]. HIV is highly stigmatized in Swaziland: people living with HIV have reported name-calling, being excluded from sources of food and water, gossip and job loss [38,39].

### Ethical considerations

The Swaziland Scientific and Ethics Committee (SEC) and the Emory University Institutional Review Board approved this study. Management and head nurses granted permission at each clinic site. Traditional leadership reviewed and authorized the study protocol at the rural study site. All participants provided written informed consent prior to beginning study procedures. Per SEC preferences we did not provide participants with any financial incentives. We did provide food, drink and childcare while they participated. The first author shared preliminary findings with the Ministry of Health, the national HIV coordinating body and clinic staff at each study site at the conclusion of fieldwork.

### Participants

The study was conducted with a clinic-based sample of pregnant women accessing antenatal care. Approximately 95% of Swazi women give birth to at least one child in their lifetime and 97% of these access antenatal care (ANC) at some point in their pregnancy [37,40]. All pregnant women receive free HIV testing, counselling and linkage to care at every antenatal visit [41]. A female Swazi research assistant (RA) approached every woman in line for services on each day of recruitment (generally Monday to Friday). Participants were eligible for the study if they were 18 years or older, receiving antenatal care that day, and spoke siSwati. Participants were eligible whether or not they were already aware of their HIV status.

### ACASI survey administration

The survey was created in English, translated into siSwati and back translated into English to check accuracy. It was piloted with a small convenience sample of urban clinic attendees (*n* = 5) using cognitive interviewing techniques [42] in which participants were asked to share their thoughts on each question as they took the survey and question wording and translation was modified as necessary to ensure clarity. The survey was self-administered in siSwati using audio computer-assisted self-interview software (ACASI). An RA assisted participants with initial demographic questions to ensure they were comfortable and then withdrew unless participants requested assistance.

Women were asked to provide a pseudonym for each sexual partner in the last 12 months. Data and analyses presented in this study are for the single most recent partner as 94% of women reported having only one sexual partner in the past 12 months.

### Measures

#### Primary predictor: transactional sex scale

In a previous study phase, we utilized cultural consensus modelling (CCM) to derive and validate a behavioural scale that can be used to measure transactional sex based on the items received from a woman’s sexual partner, and the value of those items within different models of transactional sex. CCM is a rapid, mixed-methods ethnographic technique which utilizes freelisting, rating and principle factor analysis to identify the culturally “correct” answer to a question - in this case, “What do Swazi women get, or hope to get, in exchange for sex.” Further details on CCM are provided by Romney and Weller [43,44], and a longer description of the process of scale building and validation to measure transactional sex in Swaziland is available from the work by Fielding-Miller et al. [19]. Participants were shown a list of 22 items that had been generated in a previous study phase in response to the question “What do Swazi women get, or hope to get, in exchange for sex?”. Participants were asked to identify all of the items they had received from their sexual partner in the last 12 months (Table 1). Responses were summed and weighted according to 3 different models of transactional sex (i.e., 3 different ways of valuing the 22 different items) so that each participant was assigned 3 distinct transactional sex scale scores. The three scales were then standardized using *Z*-scores to make them comparable. The scale on which a woman had the highest score was used as her transactional sex scale score as the three different models of transactional sex were highly correlated with demographic risk confounders for HIV including age and education. For more details on demographic and qualitative differences between the different models and the weighted value of different items with each model, see [19] and [20].

**Table 1.** Survey items: (1) things women hope to get in exchange for sex, (2) reasons why women agree to have sex

Transactional sex	Reasons for having sex
***Has [partner] given you any of the following items, or money for any of the following items?***	***Have you ever had sex with [partner] for any of the following reasons?***
Fun night out	Poverty
Smartphone	Spite
Airtime	Fear of violence
Clothes	Money
Toiletries	Hunger
Hairstyle	Rape or abuse
Meal at nice restaurant	Parents forced you to
Alcohol	Hope he would marry you
Basic food	Sake of your children
Takeaway	Fear he would leave
Fashionable clothes	Love
Rent	Sexual satisfaction
Fashionable shoes	To have children
Jewelry	Peer pressure
Transport	A nice lifestyle
Place to sleep	Get or keep a job
Nice lifestyle	Security
Cosmetics	So he would commit
Job	To show commitment
Things for family	Sex work
Car	Prevent infidelity
Things for child	His right as a husband
School fees	Women should submit to men
	He has a nice lifestyle
	He has high status
	So he wouldn’t cheat

#### Primary outcome: HIV status

All ANC clients receive an HIV test at every clinic visit using the Determine HIV rapid test. Positive results are confirmed using Uni-Gold HIV rapid test. Participants’ results from that day’s HIV test were recorded separately, double entered and later linked to survey data using an anonymous unique ID. Women who had tested HIV positive on a previous visit and consented to share their test results with the study team confirmed their status by showing their official clinic record card to the first author.

#### Risk mediators: social status, sexual reputation and condom use

Social status was measured using the MacArthur subjective social status scale, a globally validated instrument [45–47]. Participants were shown an image of a ladder and asked to think of it as representing where people stand in their communities. They were then asked to place themselves on the ladder according to how they considered their own social standing. Participants were told that community means many things and to think of community in a way that was relevant to them. Social status score was collapsed into a categorical variable by quartile.

Participants were asked if they had ever been called a nasty name or thought they had been called a nasty name behind their back. “Nasty name” was defined with a series of examples that implied mercenary or promiscuous sexuality (“gold-digger” or “slut”), gathered during formative focus group discussions. Responses were assessed on a 4-point Likert scale from “yes” to “no” [1].

Condom use was assessed based on condom use at last sex to reduce recall bias. Women were asked, “The last time you had sex with [partner], did you use a condom?”.

#### Control variables

Women were asked if their partner’s HIV status was positive, negative or unknown. Because Swaziland has an extremely high HIV prevalence, participants who reported that their partner was living with HIV or that they did not know their partner’s HIV status were collapsed into a single category. This was done on the assumption that in a generalized epidemic in which 1 in 4 men are living with HIV, but only 30% of these are utilizing antiretroviral therapy [33], not knowing a partner’s HIV status should create a similar motivation for condom use as knowing that a partner is living with HIV.

Intimate partner violence (IPV) was measured as a distinct construct in addition to a participant’s ability to select rape, abuse or fear of violence as a reason for having sex with a partner in the last 12 months. Violence measures were based on the World Health Organization’s violence against women instrument as adapted in similar South African studies [48,49]. For a more conservative estimate in line with previous work in southern Africa, we coded women who had experienced more than one incident in the past 12 months as having experienced IPV [50].

Relationship duration was calculated in months based on the length of time from the first time a woman reported sex with her partner to the most recent time she reported having sex. Level of education was collapsed into none, primary (through grade 7), secondary (grades 8–12) and tertiary (any university or vocational training).

#### Grouping variable: indicators of constrained agency

Survey participants were shown 27 reasons that could describe why they had agreed to have sex with a partner in the last 12 months (Table 1) and given the option to select as many as they felt were applicable (see [19] for details on item generation). Formative qualitative data and an initial exploratory factor analysis suggested that the items *poverty, spite, money, hunger, her children’s sake, violence, abuse, fear he would leave, being forced by her parents* and *hoping for marriage* were likely indicators of a single latent construct designating the experience of constrained agency which could increase risk or make exiting a relationship difficult.

We tested a measurement model using Mplus software [51] to assess whether the hypothesized cluster of reasons represented a single latent construct, which we labelled “constrained agency”. After assessing the fit of the measurement model, we examined the prevalence of constraint indicators across relationships. We created a single dichotomous grouping variable in which women were coded 0 if they were “not constrained” and 1 if they were.

### Analyses

To compare the correlation between HIV and transactional sex for women with constrained agency versus those who were not constrained, we conducted a multigroup path analysis in MPlus using a robust weighted least squares estimator (WLSMV) to account for categorical outcomes [52]. Path analysis, a form of structural equation modelling, allows the researcher to hypothesize a potential causal model based on theoretical considerations a priori, and then test the data’s fit to the proposed pathways based on observed variables[53]. Multigroup path analysis is a process through which the research tests the equivalence of these pathways across groups. Both Kline [53] and Byrne [54,55] provide excellent overviews for readers interested in further details on path analysis or multigroup structural equation modelling.

To test whether the hypothesized pathways varied significantly across the constrained and unconstrained groups, we first allowed all structural coefficients to vary freely and assessed this model for overall fit and significance of individual variables. Per recommendation by Kline, model fit was assessed based on model chi-square statistics (*p* ≥ .05), root mean square error of approximation (RMSEA) in which RMSEA ≤.05 was considered good fit, comparative fit index (CFI), and the Tucker Lewis index (TLI) [53]. For both CFI and TLI, fit indices greater than 0.95 were considered excellent fit and values over 0.90 were considered moderate fit [53].

We next constrained one structural coefficient (i.e., one proposed pathway) at a time to equality across groups and assessed how this influenced model fit. If constraining a structural coefficient to equality across groups did not result in a significant difference in model fit according to chi-square difference tests, we did not consider that proposed pathway to differ significantly across groups. If fixing a proposed pathway to equality resulted in significantly poorer fit, then that pathway was considered to differ significantly between groups. In the final model, pathways that were not significantly different across groups were constrained to equality and pathways that were significantly different across groups - i.e., those structural pathways that appeared to act differently depending on whether a woman was considered constrained or unconstrained in her relationship - were allowed to vary freely (a partially invariant model).

## Results

### Sample

A total of 406 women participated in the survey. Of these, 392 provided information on reasons why they had agreed to have sex with their most recent partner in the last 12 months and are included in this analysis. The sample represented an approximately 58% response rate, which was lower than preferable and likely influenced by a lack of financial incentive. Women who declined typically cited being in a hurry, being too busy or not being interested.

### Constrained agency as a latent construct

Poverty, spite, money, hunger, her children’s sake, violence, abuse, fear he would leave, being forced by her parents and hoping for marriage all appear to measure a cohesive latent construct with moderately good fit (RMSEA = .029, CFI = .940, TLI = .923). Each indicator loaded at greater than 0.50 with *p* ≤ 0.05.

### Outcomes by constrained agency

Summary statistics for the full sample and by constrained agency groups are shown in Table 2. A total of 115 women, 29% of the full sample, reported having sex for at least one of the reasons used to indicate constrained agency. Of the 115 women who reported at least one indicator of constrained agency, the majority reported only one. Women who reported no indicators of constrained agency were approximately 25% more likely to report that their partner was living with HIV or that they did not know their partner’s status (*p* < 0.05). Approximately 40% of women reported having used a condom at last sex, and this did not significantly differ if a woman marked any indicator of constrained agency. Six percent of women reported that they were not currently in a relationship with their most recent sexual partner, but this did not vary by indicators of constrained agency (*p* = 0.90).

**Table 2.** Summary statistics for full sample and constrained and unconstrained groups

	Full sample	Unconstrained	Constrained
Variable	% (*n*)	% (*n*)	% (*n*)
**Constrained count**			
0	70.66 (277)	70.66 (277)	29.34 (115)
1	19.90 (78)		–
2	5.10 (20)		67.78 (78)
3	2.04 (8)		17.39 (20)
4	1.53 (6)		6.96 (8)
5	0.51 (2)		5.22 (6)
6	0.00 (0)		1.74 (2)
7	0.26 (1)		0.00 (0)
			0.87 (1)
**HIV**			
Negative	66.21 (241)	64.73 (167)	69.81 (74)
Positive	33.79 (123)	35.27 (91)	30.19 (32)
**Violence**			
0–1 events last 12 months	61.89 (242)	64.13 (177)	61.89 (242)
>1 events last 12 months	38.11 (149)	35.87 (99)	43.48 (50)
**Education**			
None	3.57 (14)	3.97 (11)	2.61 (3)
Primary	24.23 (95)	25.27 (70)	21.74 (25)
Secondary	67.86 (266)	66.06 (183)	72.17 (83)
Tertiary	4.34 (17)	4.69 (13)	3.48 (4)
Partner’s HIV status*			
Negative	51.53 (202)	48.38 (134)	59.13 (68)
Positive or don’t know	48.47 (190)	51.62 (143)	40.87 (47)
**Condom at last sex**			
No	58.06 (227)	56.88 (157)	60.87 (70)
Yes	41.94 (164)	43.12 (119)	39.13 (45)
**Subjective Social Status Quartile**			
1 (1–2)	31.50 (109)	32.10 (78)	30.10 (31)
2 (3–4)	19.36 (67)	19.34 (47)	19.42 (20)
3 (5–6)	26.88 (93)	28.40 (69)	23.30 (24)
4 (7–10)	22.25 (77)	20.16 (49)	27.18 (28)
**Called a nasty name (“slut”)**			
No	69.63 (266)	71.75 (193)	64.60 (73)
Maybe no	6.28 (24)	6.32 (17)	6.19 (7)
Maybe yes	7.33 (28)	7.06 (19)	7.96 (9)
Yes	16.75 (64)	14.87 (40)	21.24 (24)
	Mean (SD)	Mean (SD)	Mean (SD)
	Range	Range	Range
**Age**	24.55 (4.99)	24.46 (5.08)	24.76 (4.77)
*N* = 392	18–42	18–42	18–37
**Relationship duration (months)**	51.02 (59.59)	50.64 (62.19)	51.93 (53.02)
*N* = 373	0.13–583.82	0.33–583.82	0.13–351.15
**Transactional sex scale (*Z*-score)**	0.14 (1.02)	0.16 (1.04)	0.10 (0.99)
*N* = 389	−1.29–4.13	−1.29–4.13	−1.29–3.31

*Significantly different between “constrained” and “not constrained” groups (*p* < 0.05).

### HIV, condom use, violence, social status and transactional sex

Table 3 shows median transactional sex *Z*-score by select outcome variables for the full sample and across constrained agency groups. Women who believed their partner to be HIV negative had significantly higher transactional sex scale *Z*-scores than women who believed their partner was living with HIV or whose status they did not know (*Z*-score 0.25 vs. 0.03, *p* = 0.04). There was also a positive association between receiving more items from a partner and higher social status (*p* = 0.01). Women who reported no indicators of constrained agency and no condom use at last sex had significantly higher transactional sex scores, an association that was not significant for the full sample or for women with constrained agency.

**Table 3.** Bivariate associations between transactional sex *Z*-score and participant HIV status, as well as secondary outcomes of interest for full sample and across groups

Variable	Full sample	Unconstrained	Constrained
	Mean (SD)	Mean (SD)	Mean (SD)
**HIV**			
Negative	0.21 (1.07)	0.24 (0.08)	0.15 (1.08)
Positive	0.07 (0.09)	0.11 (0.11)	−0.03 (0.75)
	*p* = 0.24	*p* = 0.35	*p* = 0.40
**Violence**			
0–1 events last 12 months	0.19 (1.04)	0.19 (1.04)	0.17 (1.05)
>1 events last 12 months	0.07 (1.00)	0.10 (1.04)	0.01 (0.90)
	*p* = 0.29	*p* = 0.49	*p* = 0.41
**Partner’s HIV status**			
Negative	0.25 (1.08)	0.29 (1.10)	0.16 (1.04)
Positive or don’t know	0.03 (0.95)	0.04 (0.96)	0.01 (0.91)
	*p* = 0.038	*p* = 0.046	*p* = 0.41
**Condom at last sex**			
No	**0.23 (1.05)**	**0.32 (1.08)**	0.02 (0.96)
Yes	**0.03 (0.98)**	**−0.05 (0.94)**	0.22 (1.02)
	*p* = 0.06	*p* = 0.004	*p* = 0.30
**Subjective Social Status Quartile**			
1 (1–2)	**−0.12 (0.77)**	**−0.13 (0.80)**	−0.11 (0.70)
2 (3–4)	**0.14 (0.95**	**0.04 (0.95)**	0.37 (0.92)
3 (5–6)	**0.26 (1.12)**	**0.42 (1.18)**	−0.19 (0.78)
4 (7–10)	**0.33 (1.24)**	**0.31 (1.18)**	0.37 (1.36)
	*p* = 0.010	*p* = 0.007	*p* = 0.07

Women who reported they had been called a nasty name related to their sexual reputation were significantly more likely to place themselves below the median on the subjective social status ladder than women who responded “no” or “maybe no” (67% vs. 45%, *p* < 0.001).

### Path model

**Figure 2.**
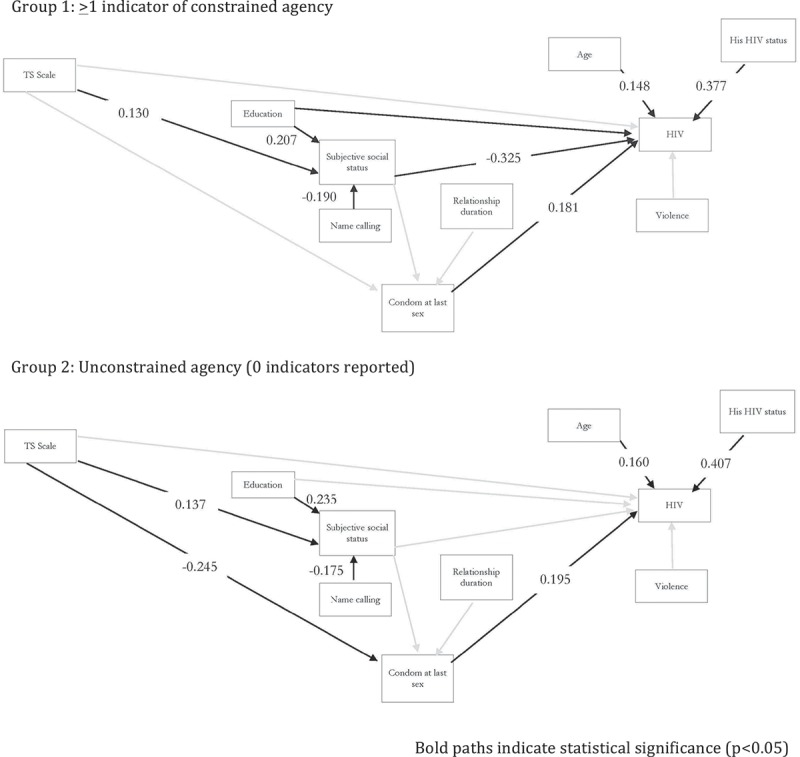
Multigroup path model diagram by unconstrained and constrained groups.

The pathways from subjective social status to HIV status and from transactional sex scale score to condom use at last sex differed significantly across groups and were freely estimated in the final model; other pathways across groups were invariant. This model had excellent global fit, with RMSEA = 0.014, CFI = 0.985, TLI = 0.978 and a chi-square value of 34.168 (df = 33, *p* = 0.41). The final model had 25 freely estimated parameters (Figure 2). According to the heuristic suggesting a 10:1 ratio of participants to parameters and Kline’s suggestion that a sample size of *n* ≥ 200 is generally sufficient for a structural model, our sample size provided sufficient power to test this model [53,56].

The direct pathway from transactional sex score to HIV status was not statistically significant for either group. For all women, receiving more items that were valued more highly was significantly associated with higher subjective social status. Each standardized increase in the transactional sex *Z*-score resulted in a 0.137-unit increase in subjective social status for women who did not experience constrained agency (*p* = 0.04), and a 0.130-unit increase for those who did (*p* = 0.04). For both groups of women, condom use at last sex was associated with a significant increase in the likelihood that a woman was living with HIV. Standardized coefficients and overall fit statistics are shown in Figure 2 and Table 4.

**Table 4.** Model fit and standardized path coefficients

*Global model fit*		
RMSEA (90% CI): 0.014 (0.00–0.057)		
**CFI: 0.985**		
**TLI: 0.978**		
**Chi-square, df (*****p*****-value): 34.168, 33(*****p***** = 0.41)**		
	Unconstrained group (***n***** = 254**)	**Constrained** (***n***** = 107**)
	***Standardized coefficient (p-value)***	***Standardized coefficient (p-value)***
**HIV**	*R*^2^ = 0.318	*R*^2^ = 0.399
Condom use at last sex	**0.195 (0.014)**	**0.181 (0.017)**
Social status*	−0.019 (0.831)	**−0.325 (0.006)**
Transactional sex scale	−0.009 (0.894)	−0.008 (0.895)
Age	**0.160 (0.024)**	**0.148 (0.026)**
Education	−0.118 (0.097)	−0.098 (0.099)
Partner’s HIV status	**0.407 (<0.001)**	**0.377 (<0.001)**
Violence	0.021 (0.749)	0.021 (0.749)
**Condoms**	*R*^2^ = 0.072	*R*^2^ = 0.051
Social status	−0.073 (0.333)	−0.074 (0.333)
Transactional sex scale*	**−0.245 (0.002)**	0.228 (0.080)
Relationship duration (months)	0.012 (0.946)	0.010 (0.946)
**Social Status**	*R^2^ = 0.123*	*R^2^ = 0.124*
Transactional sex scale	**0.137 (0.035)**	**0.130 (0.036)**
Name calling	**−0.175 (0.004)**	**−0.190 (0.004)**
Education	**0.235 (<0.001)**	**0.207 (<0.001)**

*Pathways are significantly different between “constrained” and “not constrained” groups (***p*** < 0.05).

**Note: 31 participants were missing one or more observation and were excluded for the full model.**

The indirect pathway from transactional sex to HIV status differed across groups. For women who reported any indicator of constrained agency, increased material support from a sexual partner had no significant association with condom use at last sex and higher subjective social status was associated with a 0.325 standard deviation decrease in the likelihood that she was living with HIV (*p* = 0.01). Among women who reported no indicators of constrained agency, a higher transactional sex score was associated with a decreased likelihood of condom use at last sex (standardized coefficient = −0.245, *p* = 0.01). Among these relatively unconstrained women, the pathway from subjective social status to HIV status was not significant.

## Discussion

Scoring higher on the transactional sex scale was associated with higher subjective social status for all women. It is possible that women with high social status receive more and better gifts from their partner because they are both relatively affluent. However, research in the region suggests the opposite causal pathway[7,9]: in a context in which women face 70% unemployment - compared to men’s 30% - and significant legal challenges in buying, owning or inheriting property, male partners are a primary means of accessing financial security, commercial goods, and symbolic capitol [10,12,21,35]. Women with financial resources and access to consumer goods via a high-status partner are more likely to be respected in their communities than women who do not have these things [7,31].

The negative association between subjective social status and HIV status is present only for women with constrained agency. Higher social status may decrease a woman’s risk of HIV acquisition if she feels socially empowered to negotiate sex or to leave a partner even if she is financially dependent on him. It may also enable a woman to be more selective about her partners and avoid men whom she believes may be higher risk. The reverse relationship is also possible: living with HIV may significantly decrease women’s social status, particularly for women who are already experiencing constrained relationship agency. The latter may be a product of instrumental stigma, which is engendered when a woman’s community believes that her disease makes her a potential burden who is unable to reciprocally contribute to the informal local social safety net because she lacks sufficient social or financial capital [57]. Women who report no indicators of constrained agency may be more likely to have access to social and financial resources, and consequently may not be seen as a potential burden despite their HIV status. Evidence from sub-Saharan Africa, particularly Uganda, suggests that an “ART plus” approach, in which antiretroviral therapy (ART) treatment support programmes are paired with food and livelihood interventions, have the ability to reduce instrumental stigma by improving women’s financial wellbeing and decreasing their need to rely on their neighbors, while simultaneously improving their physical wellbeing and ability to make contributions to their community [57,58].

While all relationships are driven by a range of motives, women are generally expected to be socially and emotionally dependent on their male partners. This emotional reliance - what Connell has called “cathexis” - is socially normative and a key structural driver of gendered socio-economic power disparities in southern Africa and globally [59–61]. Women who reported being called a nasty name related to their sexual reputation - local variations on “slut” or “gold-digger” - rated their subjective social status significantly lower than those who did not, likely because these nasty names describe a relationship motive in which material needs are foregrounded over social or emotional dependence. Women who explicitly reported having sex with their partner for any of the reasons which we used to determine constrained agency were also tacitly admitting that their sexual relationship was not solely motivated by affection for their male partner. These reasons - money, hunger, poverty, spite and so on - are more likely to be socially unacceptable, engendering at best pity and at worst contempt, because they violate the structure of cathexis [60,61]. For women whose femininity is perceived as noncompliant (i.e., whose motives are counter to the ways in which cathexis maintains a gendered power hierarchy), the stigma of HIV may be exacerbated by a community perception that her status is a direct - and perhaps deserved - consequence of her behavior [20]. For these women, it is essential that programming and policy efforts not stigmatize transactional relationships or reinforce gendered cultural scripts that penalize women who have sex for pleasure - or even profit - rather than affection or submission [20,23,62]. More useful are interventions that work to dismantle social structures that maintain the gendered power hierarchies that create risk, although this will require context-specific reflection from donors, researchers and programmers in the design phase and throughout implementation. While transactional sex and sex work are distinct sets of behaviours and identities, interventions with sex workers have demonstrated that community-based empowerment approaches can foster social cohesion, increase condom use and decrease the risk of HIV and other STIs without increasing stigma or reinforcing harmful gender roles [63].

In bivariate analyses, condom use at last sex did not significantly differ across the constrained and unconstrained groups, although in the full model condom use at last sex became less likely among women in unconstrained relationships with increased gift giving. As a risk pathway from transactional sex to HIV, financial support can limit agency and make condom negotiation difficult for women who are economically dependent on their partners. However, for women with less constrained agency in their relationships, the decision to forgo condoms with a sexual partner may be made based on personal preferences, and the decision is more likely to be made with agency, affection and trust. For many couples in Swaziland, southern Africa and the rest of the world, gift giving and financial support from a male partner are normative parts of courtship and relationships [13,26,64,65]. Research across the globe has shown that as intimacy and trust in a relationship increases, condom use decreases even in contexts with a high background HIV prevalence [66–70]. For women with less constrained agency, programming efforts that acknowledge women’s sexual pleasure, agency and the mutual decision-making process within a couple may be more effective than efforts predicated on the notion that women have minimal agency within their relationships.

### Limitations

This study was cross-sectional and most participants were already aware of their HIV status and so we cannot make causal inferences. Our HIV prevalence estimate was relatively low in comparisons to antenatal care sentinel surveillance data (analyses not shown), suggesting that individuals who knew their status may have avoided the study. As a result, our estimates of HIV risk may be more conservative than reality. Reported condom use at last sex is relatively high for a pregnant population and this measure may be suffering from social desirability bias. However, previous qualitative work associated with this study supports the statistical association between increased material support and decreased condom use [20].

## Conclusions

We used a behavioural scale to measure the spectrum of transactional sex within women’s relationships in Swaziland. Using a multigroup path analysis to test the hypotheses that (1) more engagement in transactional sex is associated with decreased condom use and increased subjective social status; (2) subjective social status mediates the relationship between transactional sex and HIV status and (3) these relationships are different across groups according to whether or not a woman reported any indicator of constrained agency within her relationship, we found that agency may be a more important risk factor than the amount of financial support received from a woman’s partner. For all women, receiving more valued items from a partner was associated with higher subjective social status. Among women with constrained agency, living with HIV was associated with lower subjective social status and the amount of material support was not associated with condom use. Among women who are relatively unconstrained, receiving more items was significantly associated with decreased condom use. For the latter group, gifts from a partner may partly be a manifestation of affection, and the decision to forego condoms may be as much borne of love and trust as coercion.

Programming efforts to reduce the risk of transactional sex are more likely to be successful if they focus on agency as the risk mechanism, rather than gift giving. Additionally, interventions that reinforce the gendered notion that women who engage in sexual relationships for pleasure, profit or any non-romantic motive are “non-compliant,” are likely to decrease vulnerable women’s agency and increase their risk, than protect them. Instead of focusing on whether a relationship is primarily driven by economic motives, interventions designed to mitigate the risks of transactional sex may be more successful if they focus on social factors including personal and familial poverty, access to education, and social and legal environments that necessitate women’s dependence on men for long-term socio-economic stability.
